# Optimal information disclosure strategy in the primary healthcare service market: From the perspective of signaling theory

**DOI:** 10.3389/fpubh.2022.959032

**Published:** 2022-10-28

**Authors:** Jianyue Liu, Zhiqiang Ma, Jialu Su, Bailin Ge

**Affiliations:** School of Management, Jiangsu University, Zhenjiang, China

**Keywords:** general practitioner, competency, information disclosure, signaling theory, primary healthcare

## Abstract

The promotion of general practitioner (GP) contract service is one of the key components of China's healthcare reform. We consider GPs providing primary health services with private competency information over two periods, where patients decide when to sign. Two types of GPs are considered: those with higher and lower competency. Under asymmetric information, to spur the patients' incentive to sign, the GPs can move to offer competency disclosure schemes to patients, for example, separating or pooling, through which true competency information is revealed, respectively. We investigate three scenarios, which are referred to as “separating-separating,” “pooling-separating,” and “pooling-pooling.” The results of the three scenarios yield intriguing insights into the impact of the GP's competency disclosure decisions. Findings include that GPs prefer the “pooling-separating” strategy, but patients prefer “separating-separating.” Besides, an extremely low cure rate may enable GPs to conceal some competency information. Furthermore, low-competency GPs may exaggerate their competency level for profit, but greater efforts in disclosing competency information may result in diminished benefits. Therefore, to promote the services of GPs, the core is always to improve GPs' competency.

## Introduction

A general practitioner (GP), also known as the “gatekeeper” of the primary healthcare system, is at the vanguard of comprehensive primary care services and holds a prominent position in expanding access to primary care services (1, 2). For the advantages of enhancing the residents' health along with cost reduction (3), the practice of GP service has gained wider acceptance in the primary health market worldwide (4–6). Therefore, in 2016, China proposed to establish a general practitioner system to provide contract services, ensuring that all citizens can reach comprehensive, preventive public health services and the achievement of Health China 2030 (7, 8).

Competency is the foundation for GPs' effective provision of contracted services and plays a supporting role in the construction of the hierarchical medical system. Building on the prior definitions, competency in healthcare refers to the habitual and judicious use of communication, knowledge, technical skills, clinical reasoning, emotions, values, and reflection in daily practice for the benefit of the individual and the community being served (9–12). The existing literature manifests that GPs' competency can influence demand for signing based on its impact on medical quality (13–16). However, in China, providers working in primary healthcare market with doctoral and master degrees are only 0.3 and 5.5%, along with the information asymmetry between providers and patients (17), resulting in the prejudice of patients and difficulties in promoting GP contract service in China. This problem directly leads to the decline in Chinese residents' willingness to seek medical treatment at the grassroots level. The proportion of residents who visit primary medical institutions has dropped from 57.6% in 2016 to 33.9% in 2021, putting additional strain on medical resource allocation. Since the Chinese government has been raising investment in its primary healthcare system, a better disclosure strategy of the competency, which can encourage the signing willingness of those patients, is crucial for dispersing GPs' services. This justification compels decision-makers to address GPs' competency disclosure.

Information disclosure, as one of the important tools for improving patient–provider alignment, can help patients understand professional medical information and improve the efficiency of the healthcare market (18). The reason for greater transparency is that patients value the quality of their healthcare providers (19, 20). The theory behind such activities is to improve the accountability of service providers through the disclosure of quality-of-care information (21), enabling (i) patients to seek providers with higher quality, and (ii) medical service providers to enhance quality to possess their market share. Eventually, creating a healthcare system in which social welfare is expected to increase (22). For decades, policymakers worldwide have been stepping up efforts to establish mature medical information disclosure systems (23). For example, in 2002, America launched the Hospital Quality Alliance (HQA), a national public–private organization, to announce hospital care information to the public (24). The Hospital Comparison Website was launched by the Centers for Medicare and Medicaid Services (CMS) in 2005. In 2011, British Prime Minister David Cameron pledged that the National Health Service (NHS) would publish performance data to provide citizens with modern, personalized, and long-term public services (25).

Disclosure programs undoubtedly provide the ability to provide patients with timely, relevant, and broader information, while all previous contributions ignore the issue of disclosing competency strategies among different GPs. As GPs are in advantageous positions in possessing their own competency information, some low-level GPs may take opportunistic actions to obtain more contract orders, for example, exaggerating their competency. Such activities exacerbate provider–patient conflicts and lead to the phenomenon of “bad money driving out good money” in the primary healthcare market. Accordingly, this last paper introduces the signaling theory to investigate the optimal disclosure strategy of GPs. As a fundamental tool for solving the problem of information asymmetry between two players (26), signaling theory occupies an outstanding position in various management pieces of literature, such as logistics management, entrepreneurship, human resource management, and quality management (27). A recent study of CSR governance, for example, identifies how decision-makers signal the unobservable good greenwashing of their firms to potential consumers *via* the observable CSR investments (28). Signaling theory is also vital to human resource management, where Ke and Zhu examined the signaling matching problem that happens between recruiters with private preferences information and freelancers on the decentralized freelance platform (29).

In this paper, to spur the patients into signing with the GP, combining the signaling theory, we develop a two-period game model of competency disclosure. To begin with, we assume that there exists asymmetric information between GPs and patients regarding GPs' competency. We specifically study two types of GPs based on their different levels of performance (30): those with high- (low-) competency are referred to as h-type (l-type) GPs. GPs can decide on different intensities of competency disclosure through the public report or other activities. Taking online medical services as an example, physicians can use system-generated information (education background, work experience, and number of papers) to help patients determine the quality of their service and attract more visitors (31). Also, patients are uninformed about their competency in the first stage, while they can access a signal about their competency in the second stage from the patients who have signed with GPs in stage 1 for their habit of relying on the recommendations of others (32, 33). Besides, the GP can choose one of two disclosure equilibrium strategies (separating or pooling) to distribute the competency information. Under the separating disclosing strategy, GPs are allowed to decide different disclosure intensities (e.g., publishing a different number of articles), helping patients infer the true competency. A pooling strategy permits two types of GPs to set the same disclosure intensity (e.g., similar working experience), hindering patients from extrapolating true competency. The mix of two disclosing decisions in two periods leads to three scenarios. The GP can select one of them to control the competency information. By contrasting the outcomes of the three scenarios, we intend to answer the following research questions:

(i) Which competency disclosure strategies are preferred by the GPs and by the patients?
(ii) Under the three information disclosure decisions, will low-competency GPs take deceptive behavior? Whether can they make more profits by cheating than those with high-competency?
(iii) During the process of the signing service, what factors will affect GPs' disclosure efforts and the respective benefits of GPs and patients?

The following summarizes our main results. First, preferences for the competency disclosure strategies of GPs and patients differ. GPs prefer the pooling-separating strategy while considering the maximization of their social welfare, patients prefer the separating-separating strategy. Second, when the level of primary medical facilities is too low, GPs may not be willing to announce their competency to society and even conceal some information. This finding further corroborates previous research (34). Third, to maximize their profits, low-competency GPs may inflate their competency levels to attract patients to contract with them, while increased disclosure efforts do not always result in increased benefits and can sometimes lead to additional costs. Therefore, for GPs, the core work is still to strengthen training and improve the level of competency.

The rest of this study is organized as follows. Section Methods presents the model configuration. Section Results outlines the equilibrium outcomes in various circumstances and conducts the comparison with some numerical results. Section Discussion is devoted to making some closing remarks and management insights. The proofs are included in the Supplementary material.

## Methods

### Model setup

In this section, we establish a model relying on classical signal theory, which is composed of a representative GP (“sender”) and a representative patient (“receiver”). Table 1 summarizes the notations used along with the paper. To build a model that can assist us in understanding the influence of disclosing the competency of GPs on the choices of patients, we consider a fixed continuum of patients with a total mass normalized to one that seeks treatment from *j* type GPs over two periods *i* = 1, 2. Here, *j* = *h, l* denotes high-competency GP and low-competency GP, respectively, which is determined at the outset by nature, and the GPs learn their unique type. A GP has a prior probability γ of being a high-competency type (*e*_*h*_).

**Table 1:** Notations for variables and parameters.

**Notation**	**Description**
*i*	index of the signing period, *i* = 1, 2
*j*	type of the GPs, j = h, l
ρ	discount factor
*s*_*j*_	signal's type
*a*	accuracy of the signal
*e*_*j*_	actual competency of the GP *j*
*c*_*ij*_	competency information disclosure level of the *j*-type GP at signing period *i*
*n*_*ij*_	the volume of patients having the willingness to sign with the *j*-type GP at period *i*
*N*_*ij*_	the volume of patients signing with the *j*-type GP at signing period *i*
π_*ij*_	signing profit of the *j*-type GP at signing period *i*
*H*_*ij*_	the cost of the competency disclosure activities
*PS*	patients' social welfare
*p*	signing fee
*m*	initial signing rate of the GPs
γ	the probability that the competency of the GP is high

### Equilibrium concept

We adopt the concept of perfect Bayesian equilibrium as a solution (35). In brief, given the prior belief γ about the type of GP, the patient fine-tunes the patient's belief in light of Bayes' rule and optimally reacts to the GPs' observed disclosure actions. The GP optimally determines their competency information disclosure level according to the patient's posterior belief and reactions.

Specifically, patients in the first stage can only obtain a prior probability about the GP's competency, but patients in the second stage have access to a posterior probability. Let *e*_*j*_ (*j* = *h, l*) denotes the true competency of GP *j*, while *s*_*j*_ characterizes the signal about the competency obtained by the patients in period 2 from the first-stage patients. Using *a* to depict the accuracy of the signal from the early patients, that is, Pr(*s*_*h*_|*e*_*h*_) = Pr(*s*_*l*_|*e*_*l*_) = *a*. Such information structure has been widely applied in modeling incomplete information in previous research (36, 37).

Hence, the Bayesian-updated probabilities of competency based on the comments *s*_*h*_ and *s*_*l*_ are as follows:


$$\begin{array}{l} \;Pr(e_{h}|s_{h})=\frac{a\gamma }{Pr(s_{h})},\;\mathrm{Pr}(e_{l}|s_{h})=\frac{\left(1-a)(1-\gamma \right)}{Pr(s_{h})},\mathrm{Pr}(e_{h}|s_{l})\\ =\;\frac{\left(1-a)(\gamma \right)}{Pr(s_{l})},\mathrm{Pr}(e_{l}|s_{l})=\frac{a(1-\;\gamma )}{Pr(s_{l})},\; \end{array}$$

where Pr(*s*_*h*_) = *aγ*+(1−*a*)(1−γ) and Pr(*s*_*l*_) = γ(1−*a*)+*a*(1− γ).

The equivalent competency *e* depending on the signals are as follows:


$$\begin{array}{l} \;E[e|s_{h}]=e_{l}\mathrm{Pr}(e_{l}|s_{h})+e_{h}Pr(e_{h}|s_{h}),E[e|s_{l}]=e_{l}\mathrm{Pr}(e_{l}|s_{l})\\ +\;e_{h}Pr(e_{h}|s_{l}).\; \end{array}$$

**Figure 1:**
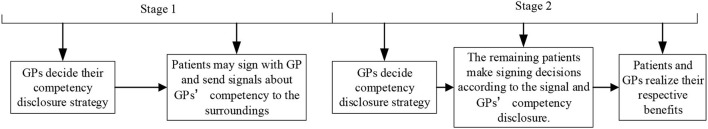
Decision sequence.

As in the previous literature (28, 38), our study only concentrates on pure-strategy equilibria in this paper, that is, separating equilibrium and pooling equilibrium. Under the premise of the same signing fee *p*, in the separating equilibrium, different types of GPs will choose different competency information disclosure efforts, such as the different number of published articles, work, and study experience, to assist patients in accurately determining the type of GPs; in a pooling equilibrium, two types of GPs choose to publish the same information. For example, if two types of GPs publish the same number of papers, the same information makes it impossible for patients to determine the true type of GPs, only the prior probability. Based on the preceding analysis, and given that this paper is an intertemporal decision-making problem, Figure 1 depicts the sequence of each player's decisions. We shall consider three disclosure scenarios:

Separating-separating equilibrium: GPs who own different level of competency optimizes their respective profit by choosing different disclosure strategies over time $\overset{⇀}{c_{1j}}$, $\overset{⇀}{c_{2j}}$, *j*∈{*h, l*}. By assessing the separating information about GPs' competency in the stage 1, all patients can perfectly infer the true competency of each GP by analyzing the information in both signing stages.

Pooling-separating equilibrium: There could be a pooling equilibrium in period 1, in which the two types of GPs disclose the same information about their competency, that is, $\overset{\sim }{c_{1}}=c_{1j}$, whereas the information disclosed in period 2 differs, $\overset{\sim }{c_{2j}}$, *j*∈{*h, l*}. Hence, patients cannot distinguish the GP's competency level in the first signing stage, but can exactly deduce the GPs' competency in the second signing stage.

Pooling-pooling equilibrium: Different types of GPs disclose the same information in each signing stage, that is, $\hat{c_{1}}=c_{1j}$, $\hat{c_{2}}=c_{2j}$ *j*∈{*h, l*}. Therefore, patients cannot tell the GP's true type in the first period, while in the second signing stage, prospective patients can get a signal regarding competency from the patients in the first stage. Thus, the patients can renew their expectation about GP's competency taking account of this signal.

### Patients' utility

As for the patients, they can choose to sign with the GP immediately or postpone the signing until the second stage.

To model a patient's choice, we assume the utility of a patient who chooses GP *j* depends on the competency and signing fee. Given the signing bonus *p* and the perceived information *c*_1*j*_ that the GPs *j* diclose, the patients' expected utility from signing the contract with the specific GP in the first stage is as follows:


(1) $$\begin{array}{l} U_{1}=v+c_{1j}+E\left[e_{j}\right]-p, \end{array}$$

where the *E*[*e*_*j*_] = γ* e*_*h*_+(1−γ) *e*_*h*_ = μ.

Similar to Bisceglia et al. (39), *v* is the patient's private valuation of the medical service provided by the specific GP *j*. It describes the initial signing willingness of patients with those specific GPs. Due to that there exists heterogeneity of signing intention among different individuals, this study supposes it is uniformly distributed over [0, 1]. When their valuations of *j* GP are higher than $\overset{⇀}{v_{j}}$, a total of $n_{1}=1-\overset{⇀}{v_{j}}$ patients will have the willingness to sign with the *j* GP in the first phase. The threshold $\overset{⇀}{v_{j}}$ divides patients who seek the GPs into the early and late signing stage. This paper is a classical intertemporal selection problem, which requires patients to trade off costs and benefits at different points in time (40). We introduce the discount factor ρ > 0, and the value of threshold $\overset{⇀}{v_{j}}$ can be accessed by solving *U*_1_ > ρ*U*_2_. Because of information asymmetry, the true value of *e* is the GPs' intimate message about their competency, which is also not originally known by patients who merely have access to the prior distribution *E*[*e*_*j*_]. Besides, the utility of the patients increases with the competency disclosure level *c*_1*j*_ and decreases with the signing fee *p*.

Those patients with valuations below $\overset{⇀}{v_{j}}$ would delay signing to the second period. At this moment, they get additional competency signals as they receive evaluation information from the patients who have signed with the specific GP in stage 1. Hence, the patients' expected utility in stage 2 is as follows:


(2) $$\begin{array}{l} U_{2}=v+c_{2j}+E\left[e|s_{j}\right]-p, \end{array}$$

Here, *E*[*e*|*s*_*j*_] portrays the patients in the second stage can know the posterior quality probability of GPs' competency level, because they can access a signal about competency by reviewing comments from the patients who have signed with GPs in stage 1. The corresponding information set $I_{t}=\left(I^{1},I^{2}\right)$ depicts the information available to the patient in three scenarios *t* = 1, 2, 3, where *I*^1^ denotes the perceived information of patients in period 1, and *I*^2^ represents that in period 2. Notice that *I*_1_ = (*e*_*j*_, *e*_*j*_), *I*_2_ = (*E*[*e*], *e*_*j*_), *I*_3_ = (*E*[*e*], *s*_*j*_). In this phase, the remaining patients with a non-negative utility will consider signing the contract with the *j* GP, that is, *U*_2_> 0, which formulates the minimum threshold of the valuation and the potential volume of the contracts $n_{2}=\overset{⇀}{v_{j}}-\max\left\{0,\;vj\right\}$.

To emulate the perspective of society as a whole, or equivalently that of a decision-maker charged with preserving societal interests, we define patients' social welfare as the sum of the expected surplus in both periods obtained by all patients, as provided by the following:


(3) $$\;PS_{1}=\sum Pr(e_{j})\int_{\vec{v_{j}}}^{1}\{v+E[e|I^{1}]+c_{1j}-p\}dv,$$

(4) $$\begin{array}{l} PS_{2}=\sum Pr(e_{j})\int_{\underset{\max}{\left\{0,\right\}}}^{\vec{v_{j}}}\{v+E[e|I^{2}]+E[c_{2j}|I^{2}]-p\}dv, \end{array}$$

or


(5) $$PS_{2}=\sum Pr(s_{j})\int_{\underset{\max}{\left\{0,\right\}}}^{\vec{v_{j}}}\{v+E[e|I^{2}]+E[c_{2j}|I^{2}]-p\}v,\;j\in \left\{h,l\right\}.$$

Patients would consider the sum of welfare in signing stage 1 and the expected welfare in stage 2 when making their respective signing decisions. The total patients' social welfare is *PS* = *PS*_1_+ρ*PS*_2_.

### GPs' objectives

Of note, although a significant number *n*_*ij*_ of patients might have the willingness to opt for *j* GPs, not all of them end up signing with them. Hence, they need to decide their competency information disclosure levels *c*_*ij*_ to attract patients to sign with them, maximizing their signing profits. Based on this hypothesis, the actual number of people signing up with *j* GP is as follows:


$$\begin{array}{l} N_{ij}\left(c_{ij}\right)=n_{ij}^{*}m^{*}\left(1+c_{ij}\right). \end{array}$$

In this setting, *m*∈(0, 1) represents the initial signing rate of the patients, which is affected by the competency disclosure level *c*_*ij*_. For simplicity, denote by *r* cure rate of *j* GP and by *m* the resulting patients' initial signing rate with *j* GP, with *m* = *f*(*r*), where *f*() is an increasing function. Assuming a monotone increasing relationship between cure and initial contract rate, we can express the total signing volume as a function of initial contract rate *m* and competency disclosure level *c*_*ij*_.

We consider the following function for the cost of competency disclosure activities:


$$\begin{array}{l} H_{ij}\left(c_{ij}\right)=\;\frac{1}{2}{c_{ij}}^{2}. \end{array}$$

This functional form assumes that the cost of each GP's disclosure actions is strictly growing and convex.

Citing the functional forms given above, the *j* GP's optimization problem in the first period is as follows:


(6) $$\begin{array}{l} \pi_{1j}=\underset{c_{1j}}{\max}\left\{{\left(1-\overset{⇀}{v_{j}}\right)}^{*}p^{*}\left(m^{*}\left(1+c_{1j}\right)\right)-\frac{1}{2}{c_{1j}}^{2}+\rho \pi_{2j}\right\}, \end{array}$$

which means that GP obtains a benefit from the signing fee *p*, the total volume of the contracts *N*_*ij*_ that depend on the competency disclosure level *c*_1*j*_, and pays the cost of investment in competency disclosure *H*_*ij*_ at that period. Besides, in the first signing period, GP has to consider the discounted profit in the stage 2, in which discount factor is labeled as ρ.

Consistent with π_1*j*_, we model the profit to the GP of type *j* in the second stage as follows:


(7) $$\begin{array}{l} \pi_{2j}=\underset{c_{2j}}{\max}\left\{{\left(\overset{⇀}{v_{j}}-\max\left\{0,\right\}\right)}^{*}p^{*}\left(m^{*}\left(1+c_{2j}\right)\right)-\frac{1}{2}{c_{2j}}^{2}\right\}. \end{array}$$

## Results

This section derives the optimal competency disclosure decisions for three scenarios.

### Separating-separating equilibrium

In this case, GPs choose different intensity levels of competency disclosure *c*_*ij*_, which means that all patients can distinguish the true competency level of the medical services *e*_*j*_ by analyzing the information. The GP's optimization is as follows:


$$\begin{array}{l} \;\vec{\;\pi_{1j}}={\;}_{{}_{\vec{c_{ij}}}}^{\max}\{\left(1-\vec{v_{j}}\right)pm\left(1+\vec{c_{1j}}\right)-\frac{{\vec{c_{1j}}}^{2}}{2}\\ +\rho \left(\left(\vec{v_{j}}-\left(p-e_{j}-\vec{c_{2j}}\right)\right)mp\left(1+\vec{c_{2j}}\right)-\frac{{\vec{c_{2j}}}^{2}}{2}\;\right)\}. \end{array}$$

The GP's strategies and profits are summarized in the following propositions.

**Proposition 1**. *In the case of separating-separating equilibrium, the competency information disclosure level of the* *j* *GP in the first period is given by*


(8) $$\begin{array}{l} \overset{⇀}{c_{1h}}=\frac{-b_{1}+\sqrt{c_{1}}}{1+\frac{2mp\left(-1+2mp\right)}{1+mp\left(-2+\rho \right)-\rho }+\frac{m^{2}p^{2}\left(-1+2mp\right)\rho }{{\left(1+mp\left(-2+\rho \right)-\rho \right)}^{2}}}, \end{array}$$

*and*


(9) $$\vec{c_{cc}}=\frac{\begin{array}{c} mp(-m^{2}p^{3}{\left(-2+\rho \right)}^{2}+2{\left(-1+\rho \right)}^{2}\\ +2mp^{2}(2+m(4-3\rho )-3\rho +\rho^{2})+A_{11}) \end{array}}{\begin{array}{c} {\left(-1+\rho \right)}^{2}+2m^{3}p^{3}(-4+3\rho )+m^{2}p^{2}(12-11\rho +\rho^{2})\\ -2mp(3-4\rho +\rho^{2}) \end{array}},$$

*respectively, where the expression of* *c*_1_, *b*_1_, *and* *A*_11_ *is given in* *Supplementary material*.

*The corresponding second-period information disclosure level is*


(10) $$\begin{array}{l} \overset{⇀}{c_{2h}}=-\frac{mp\left(-1+\rho +\overset{⇀}{c_{1h}}\right)}{1+mp\left(-2+\rho \right)-\rho }, \end{array}$$

(11) $$\vec{c_{2l}}=\frac{\begin{array}{c} mp(m^{2}p^{2}(8+p(-2+\rho )-6\rho )+{\left(-1+\rho \right)}^{2}\\ -mp(-1+\rho )(-6+p+\rho )+A_{12}) \end{array}}{\begin{array}{c} {\left(-1+\rho \right)}^{2}+2m^{3}p^{3}(-4+3\rho )+m^{2}p^{2}(12-11\rho +\rho^{2})\\ -2mp(3-4\rho +\rho^{2}) \end{array}},$$

*respectively, where the expression of* *A*_12_ *is also shown in* *Supplementary material*.

*Proof*. See Supplementary material.

By substituting the above equilibrium outcomes of competency disclosure level into the objective functions, we can calculate the respective revenue of *j* GP in each stage $\overset{⇀}{\pi_{ij}}$,*i*∈{1, 2}, *j*∈{*h, l*}, as well as the gross revenues $\overset{⇀}{\pi_{j}}$. Here, we can infer that the total profit of different GPs is equal, that is, $\overset{⇀}{\pi_{h}}=\overset{⇀}{\pi_{l}}$. This outcome illustrates that to distinguish themselves from the low-competency GP, *h* GPs have to increase their disclosure level to the point where the *l* GP does not want to emulate without affecting *h* GP's earnings. However, this decision leads to profit loss of *h* GPs.

### Pooling-separating equilibrium

In this scenario, the GPs determine the same disclosure level in the first period and different disclosure level in period 2. Recall that the profit function is given by the following:


$$\begin{array}{l} \;{\tilde{\;\pi }}_{1j}={\;}_{{}_{\tilde{c_{1}},\tilde{c_{2j}}}}^{\max}\{(1-\tilde{v})pm(1+\tilde{c_{1}})-\frac{\overset{\sim \;2}{c_{1}}}{2}\\ +\;\rho ((\tilde{v}-(p-e_{j}-c_{2j}))pm(1+c_{2j})-\;\frac{c_{2j}{}^{2}}{2})\}. \end{array}$$

The following proposition gives, albeit not in an explicit form, the optimal strategies in the two periods.

**Proposition 2**. *In the case of pooling-separating equilibrium, the competency information disclosure level of the* *j* *GP in the first period is given by*


(12) $$\tilde{c_{2h}}=\frac{-mp(mp-\tilde{v}-e_{h}-1)+\sqrt{\begin{array}{c} mp(e_{h}-e_{l})(2-2mp-2mp^{2}\\ +2mp\tilde{v}+mpe_{l}+mpe_{h}) \end{array}}}{\left(1-2mp\right)},$$

*and*


(13) $$\begin{array}{l} \overset{\sim }{c_{2l}}=\frac{-mp\overset{\sim }{v}-mpe_{l}-mp+mp^{2}}{\left(2mp-1\right)}, \end{array}$$

*respectively, where the expression of* $\overset{\sim }{v}$ *is given in* *Supplementary material**. Then, the GP's first-period disclosure intensity is the positive root of the following polynomial:*


$$\begin{array}{l} \tilde{\pi }={\;}_{\tilde{c_{1}}}^{\max}\{(1-\tilde{v})pm(1+\tilde{c_{1}})-\frac{1}{2}{\tilde{c_{1}}}^{2}+\rho (\gamma {\tilde{\pi }}_{2h}+(1-\;\gamma ){\tilde{\pi }}_{2l})\}. \end{array}$$

*By substituting* $\overset{\sim }{c_{1}}$ *into the following profit functions can obtain the respective benefits of each*


$$\begin{array}{l} \;{\tilde{\pi }}_{h}=(1-\tilde{v})pm(1+\tilde{c_{1}})-\frac{1}{2}\overset{\sim \;2}{c_{1}}+\rho {\tilde{\pi }}_{2h},{\tilde{\pi }}_{l}\\ =(1-\tilde{v})pm(1+\tilde{c_{1}})-\frac{1}{2}\overset{\sim \;2}{c_{1}}+\;\rho {\tilde{\pi }}_{2l} \end{array}$$

*Proof*. See Supplementary material.

As explained in Supplementary material, the root of $\overset{\sim }{c_{1}}$ cannot be analyzed without resorting to numerical simulations. For the rest, we will illustrate our results numerically with parameter values.

### Pooling-pooling equilibrium

Given that in this scenario, the GPs of different types disclose the same level of competency information in each period, the objective function becomes


$$\begin{array}{l} \;\hat{\pi }={\;}_{\hat{c_{1}},\hat{c_{2}}}^{\max}\{(1-\hat{v})pm(1+\hat{c_{1}})-\frac{1}{2}{\hat{c_{1}}}^{2}+\rho (Pr(s_{h})\pi_{2j}(s_{h})\\ +\;Pr(s_{l})\pi_{2j}(s_{l}))\}, \end{array}$$

where π_2*j*_(*s*_*h*_) and π_2*j*_(*s*_*l*_) represent the benefits obtained by the GP when prospective patients infer the GP's type by assessing the signal about the GP from early sufferers. That is, according to this signal, profits will vary as these patients update their expectations about the GPs' type.

The GP's optimal disclosure strategies in each period when the patients receive different signals are summarized in the following proposition.

**Proposition 3**. *In the case of pooling-pooling equilibrium, the competency information disclosure level of the* *j* *GP in the second period is given by*


(14) $$\hat{c_{2}}(s_{h})=\frac{\begin{array}{c} mp(-1+a+p-ap+\gamma -2a\gamma -p\gamma +2ap\gamma +\\ \left(-1+a+\gamma -2a\gamma )\hat{v}+(-1+a+\gamma -a\gamma )e_{l}-a\gamma e_{h}\right) \end{array}}{\left(-1+2mp)(1-\gamma +a(-1+2\gamma )\right)},$$

*and*


(15) $$\hat{c_{2}}(s_{l})=\frac{\begin{array}{c} mp(a-ap+\gamma -2a\gamma -p\gamma +2ap\gamma +(a+\gamma -2a\gamma )\hat{v}+\\ \left(a-a\gamma )e_{l}+\gamma e_{h}-a\gamma e_{h}\right) \end{array}}{\left(-1+2mp)(-\gamma +a(-1+2\gamma )\right)},$$

*respectively, where the expression of* $\hat{v}$ *is given in the* *Supplementary material**. Then, the GP's first-period disclosure intensity is the positive root of the following polynomial:*


$$\begin{array}{l} \;\hat{\pi }={\;}_{\hat{c_{1}}}^{\max}\{(1-\hat{v})pm(1+\hat{c_{1}})-\frac{1}{2}{\hat{c_{1}}}^{2}+\rho (Pr(s_{h})\pi_{2j}(s_{h})\\ +\;Pr(s_{l})\pi_{2j}(s_{l}))\}. \end{array}$$

*Proof*. See Supplementary material.

Similar to the previous, since the explicit solution of the first stage cannot be solved, we obtain the optimal equilibrium strategy of the first stage through numerical simulation.

### Numerical analysis

In the previous sections, we characterize the equilibrium solutions for the high- and low-competency GPs under the three scenarios. In this part, the numerical simulation is conducted to identify the effects of the modeling parameters, including signing fee *p*, initial signing rate *m*, and the competency parameters *e* on the equilibrium intensity of competency disclosure *c*, profits π, and patients' social welfare *PS*, among the above strategies. We start by describing the methodology used and, next, show the outcomes.

**Figure 2:**
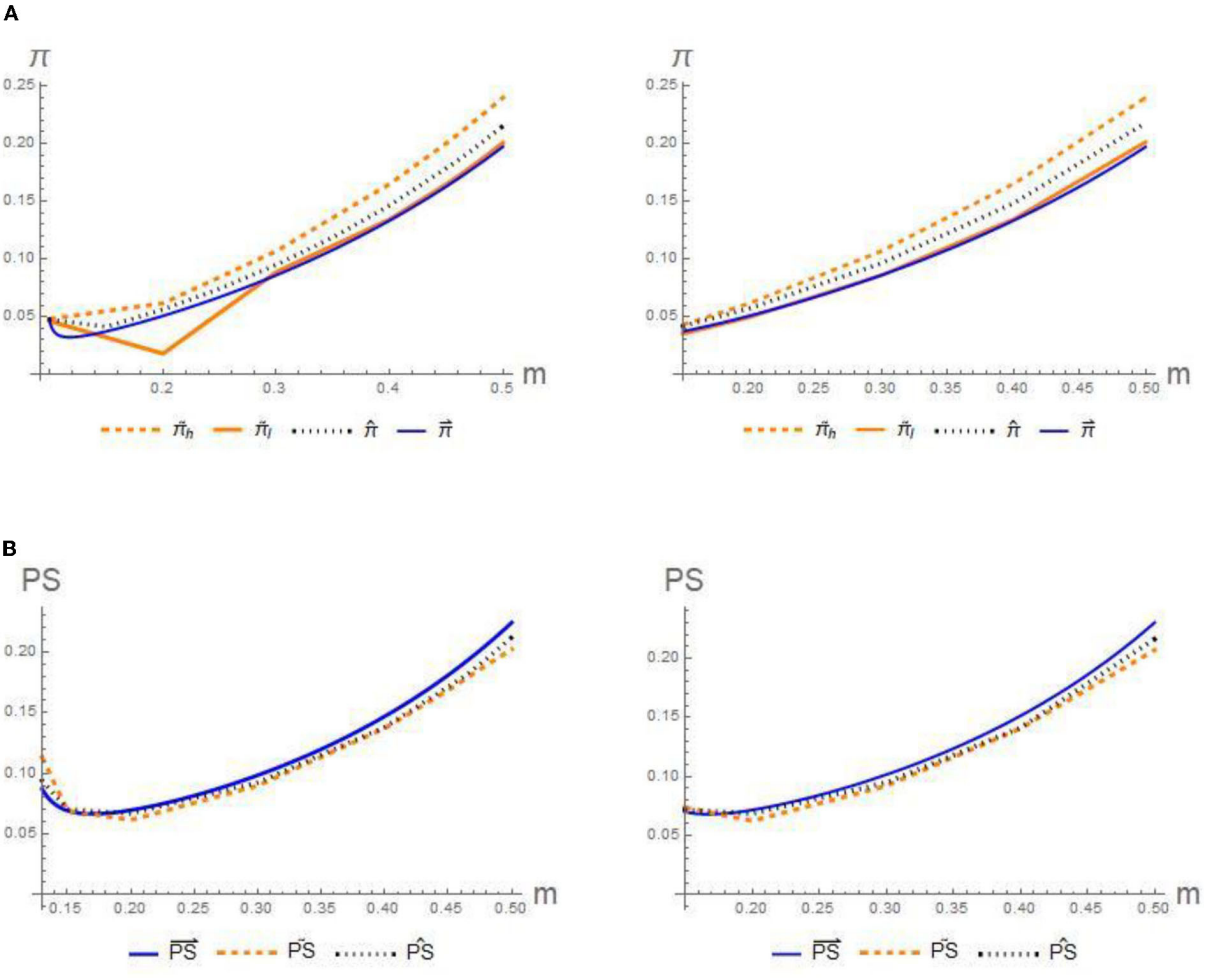
Impact of initial signing rate on the **(A)** profits π and **(B)** patients' social welfare *PS*.

The first step is to select the standard for determining the most efficient equilibrium result. Then, we consult the previous literature to determine the parameter values for this paper, and we use the Mathematica software to simulate the trajectories of the results obtained in the preceding subsections. Finally, we examine the responses of the equilibrium strategies to the key parameters using the sensitivity test. During this process, we also vary relevant parameters within a range of ±15% to check whether the qualitative outcomes of the equilibrium strategies are robust to the model calibration (41). Here, we only present the partial results of the robustness test for the parameter γ in Figure 2, but we verify that the robust tests of the rest parameters also hold.

We cite the concept of lexicographically maximum sequential equilibrium (LMSE) as the standard for choosing the most efficient equilibrium outcome (42). According to this principle, the revenue of players who have the most incentive to reveal their true type will be viewed as the criteria for judging the optimal outcome, that is, *h* GP. Specifically, the pooling equilibrium strategy would be selected if the high-competency GP can gain more profits under the pooling equilibrium than under the separating strategy.

We performed various numerical simulations by varying the different parameter values around the following benchmark: *p* = 0.8, ρ = 0.95, *a* = 0.7, *e*_*h*_ = 0.09, *e*_*l*_ = 0.01, *m* = 0.5, γ = 0.4.

The results are shown in a series of charts. In each diagram, we plot the variation of equilibrium outcomes that change one parameter, while the rest retain at their base case level.

### Impact of *m* on equilibrium outcomes

Using the previous baseline values, we illustrate in figures the influence of *m* on the equilibrium disclosure intensity, profit, and patients' social welfare, respectively. To meet the concavity of the functions and avoid trivial results, that is, 2*mp*−1 < 0, the range of values of *m* is finite (see the Supplementary material for more details).

**Conjecture 1**. *Figures 2A*,*B* *presents that for three scenarios, the profits of GPs and patients both increase with the signing rate, whereas the preferences for optimal disclosure strategies of each deviate. According to LMSE, the high-competency GP has an incentive to choose the pooling-separating strategy, while the patients prefer the separating-separating strategy. Formally:*


$$\tilde{\pi_{h}}>\hat{\pi }>\vec{\pi },\vec{PS}>\hat{PS}>\;\tilde{PS}.$$

To interpret this conjecture, the signing rate *m* is positively correlated with the cure rate of the GPs. Intuitively, the signing rate raises prospective patients' utility, stimulating the total demand, which finally brings about higher profits and higher patients' welfare. As such, the increased cure rate leads to a win–win outcome for the GPs and patients.

**Figure 3:**
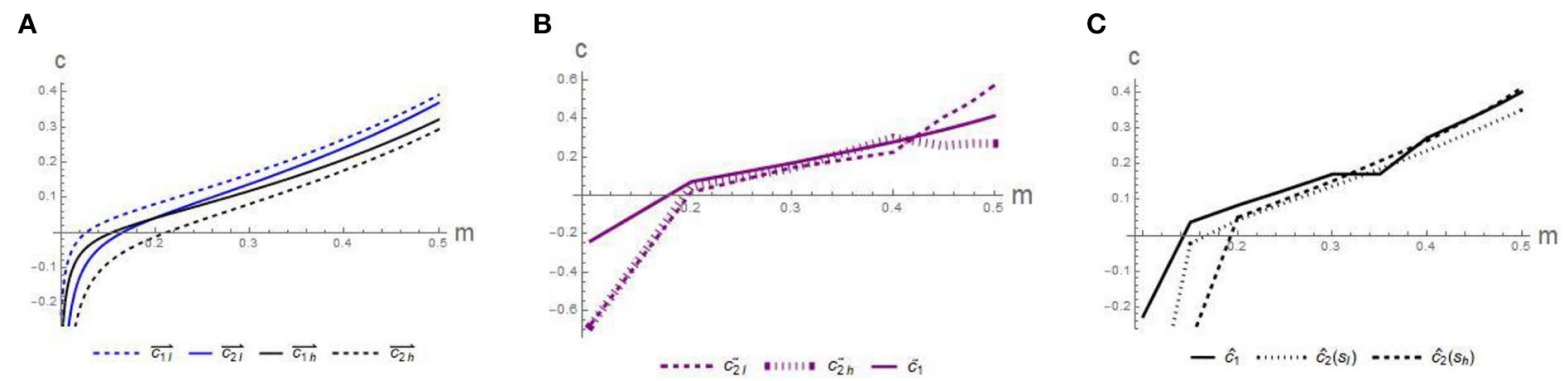
Impact of initial signing rate on the disclosure intensity *c* in scenario: **(A)** separating-separating, **(B)** pooling-separating, and **(C)** pooling-pooling.

**Conjecture 2**. *Comparing the trajectories in* *Figures 3A*–*C**:*

1. *The equilibrium disclosure level* *c* *increases with the initial signing rate* *m*.
2. *An extremely low cure rate leads to the phenomenon of concealing competency*.
3. *Implementation of the competency disclosure may lead to more costs, resulting in a certain loss of revenue*.

Figure 3 reveals that first, in all scenarios, the equilibrium disclosure level small fluctuates, but generally increases with the signing rate *m*. This illustrates the importance of increasing the contracting rate, which requires increased investment in medical equipment, since the initial contracting rate is positively correlated with the cure rate. Second, we find that the value of *c* is negative when the *m* is too low. This finding indicates that an extremely low cure rate may enable GP to conceal some competency information, which further confirms the previous findings. Third, combined with Figure 2, in the pooling-separating equilibrium strategy, although the information disclosure level of *h* GP is less than that of *h* GP in the separating-separating equilibrium strategy, the income of *h* GPs is higher than that of the separating-separating equilibrium strategy, which means that the implementation of the separating-separating strategy leads to more disclosure costs, resulting in a certain loss of revenue. Therefore, it is necessary to establish necessary information disclosure mechanisms, such as information sharing platforms, to reduce the cost of disclosure. In the long run, it is possible to realize the supervision of the competency of GPs and encourage them to continuously improve their competency, prevent the occurrence of bad money driving out good money, and promote the growth of the entire GP medical team and the improvement of the comprehensive medical level.

#### Impact of *p* on equilibrium outcomes

**Figure 4:**
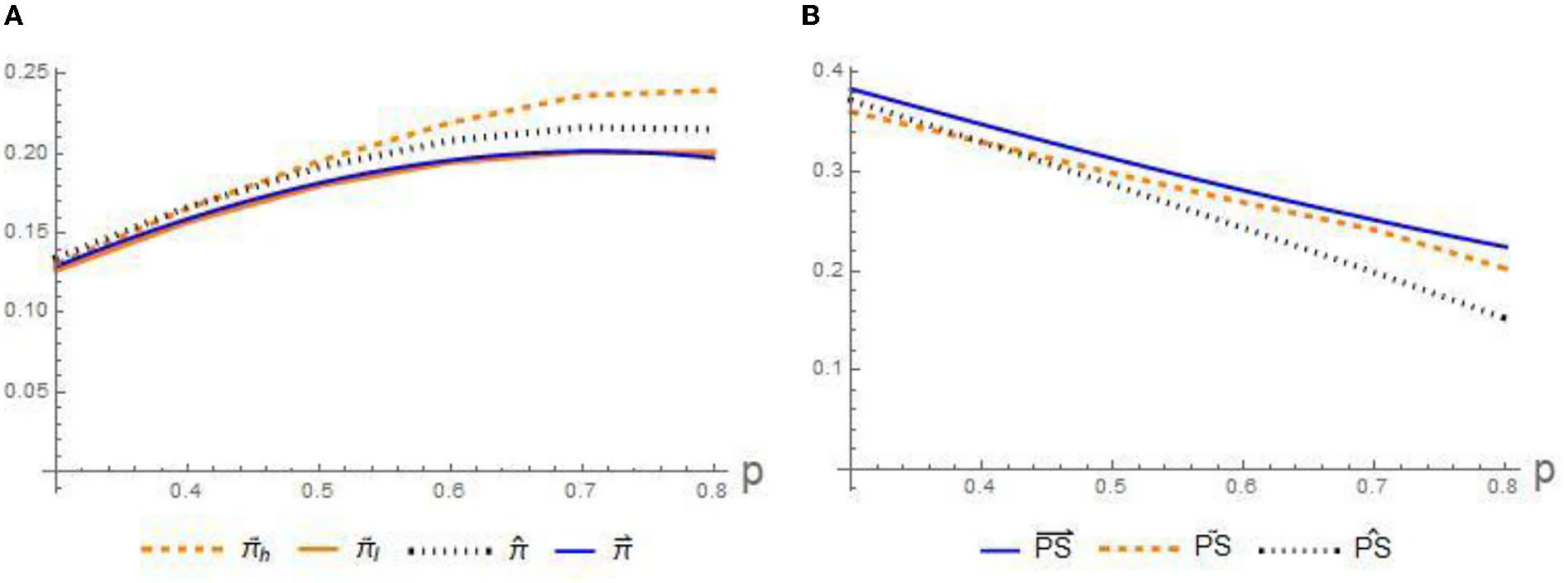
Impact of signing fee on the profits on **(A)** profits π and **(B)** patients' social welfare *PS*.

**Figure 5:**
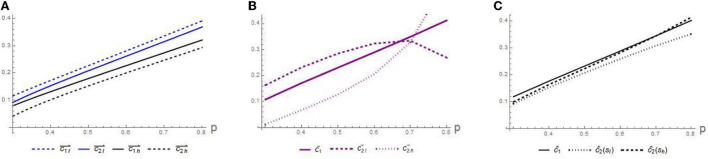
Impact of signing fee on the disclosure intensity *c* in scenario: **(A)** separating-separating, **(B)** pooling-separating, and **(C)** pooling-pooling.

Based on the previous parameter values and *m* = 0.5, the following part portrays the influence of the signing fee *p* on the equilibrium disclosure level, profit, and patients' social welfare in Figures 4, 5, respectively.

**Conjecture 3**. *Figures 4A*,*B* *presents that for three scenarios, the profits of GPs increase with the signing fee* *p, whereas the welfare of the patients decreases. Besides, the preferences of both are in line with those in Conjecture 1*.

The statement for Conjecture 3 illustrates how the different signing fee *p* affect the benefits. The above claim indicates that the signing fee *p* acts as an incentive for the GPs to increase their effort to attract more residents signing with them and at the same time results in a loss of welfare for these patients. Therefore, given the unattractive benefits of patients in this situation, it is necessary for the government and relevant departments to strengthen the subsidy for contracting fee *p*, reducing the contracting costs of residents. Only by this way, we can promote more patients to sign with those specific GPs.

**Conjecture 4**. *Comparing the trajectories in* *Figures 5A*–*C**:*

1. *The equilibrium disclosure level* *c* *increases with the signing fee* *p*.
2. *Similar to* *Figure 3A*, *l* *GP might exaggerate their true competency to maximize profits, resulting in a lack of trust between patients and GPs*.

For GPs, once a patient signs up with them, it will bring them benefits. For profit-seeking purposes, *l* GPs may exaggerate their competency and send false signals. However, patients' trust levels decreased when competency signals were concentrated at higher levels. In addition, increasing disclosure practices also incurs additional costs. Such intuitions induce the instantaneous profit of *h* GP is still higher than that of *l* GP. Therefore, for GPs, daily normalized information disclosure is necessary, but the investment in their competency level cannot be ignored.

## Discussion

In this research, we compare the effects of three competency disclosure strategies that have seldom been considered in previous research. By considering patients' behaviors, this research tries to investigate the GP's disclosing strategy and provide more managerial insights into the interaction between patients and the GP. To the best of our knowledge, this research that draws on signaling theory to explore GPs' competency disclosure decisions has never been brought to light in the literature.

Based on our analysis, we derive the following results and managerial insights. The main is summarized as follows. First, through numerical simulations, we can find that, for the *h* GP, it is always optimal to implement a pooling-separating disclosure strategy for profit in this scenario max. While for the patients, the social welfare of the patients will deviate due to the impact of the contract fee. Therefore, for the health department, relevant institutions need to strengthen the subsidy for signing fees to increase the utility of these patients, and in the long run, such activities would ensure the promotion and application of GP services in society. Second, increased disclosure efforts do not always result in increased overall benefits and may sometimes result in additional costs. Accordingly, for GPs, the core work is still to strengthen training and improve the level of their competency. Third, for pursuing more profits, *l* GPs may exaggerate their level of competency, which will exacerbate distrust between doctors and patient. Hence, the establishment of a standardized medical information sharing platform can not only reduce the cost of disclosure, but also improve the trust level of patients, and promote a win–win situation for patients and GPs. Finally, extremely low cure rate may allow GPs to withhold some competency information. This also requires policymakers to invest more in primary health services, medical equipment, etc., upgrading the hardware of primary medical and health institutions, and changing patients' prejudice that primary medical and health institutions are poor.

There are certain limitations to our study. In this paper, we make some simplifying assumptions that are worth investigating in future investigations. First, how would the results change if the residents' signing willingness affected the evolution of the GP's competency? This question is of interest since signing willingness can affect the GPs' perception of career satisfaction. Second, we can use empirical or data-driven methods to calibrate model parameters and make the theories in this study more applicable.

## Data availability statement

The original contributions presented in the study are included in the article/Supplementary material, further inquiries can be directed to the corresponding author.

## Author contributions

ZM and JL: conceptualization, literature review, writing, and editing. JL and JS: methodology. JL: calculation. BG: checking. All authors have read and agreed to the published version of the manuscript.

## Funding

This research is funded by the National Natural Science Foundation of China (71974082).

## Conflict of interest

The authors declare that the research was conducted in the absence of any commercial or financial relationships that could be construed as a potential conflict of interest.

## Publisher's note

All claims expressed in this article are solely those of the authors and do not necessarily represent those of their affiliated organizations, or those of the publisher, the editors and the reviewers. Any product that may be evaluated in this article, or claim that may be made by its manufacturer, is not guaranteed or endorsed by the publisher.
