# Supplementary material for: Optimal information disclosure strategy in the primary healthcare service market: From the perspective of signaling theory
Source: Front Public Health. 2022 Oct 28;10:959032. doi: 10.3389/fpubh.2022.959032 (PMC9650283; doi:10.3389/fpubh.2022.959032)
Supplement: Supplementary file 1 [file Data_Sheet_1.pdf]

Supplementary Material

Appendix: Proof of Propositions

Proposition 1: In this case, GPs choose different intensity levels of competency disclosure, which means that all patients can distinguish the true competency level of the medical services e_j by analyzing the information. To calculate the optimal disclosure strategies, the problem can be solved backward by computing the GPs' second-stage competency disclosure decisions.

First, we need to solve the threshold $\overrightarrow{v_j}$, above which patients would have the willingness to sign with the j GPs in the first period. Consequently, the total volume of signing contracts in the first stage is given by $n_{1j} = 1 - \overrightarrow{v_j}$. Otherwise, the patients will consider signing the contract in the second stage if they have a non-negative utility

$$v + c_{2j} + e_j - p > 0,$$

which yields $\underline{v_j} > p - e_j - c_{2j}$. Then, we can aclculate the total volume of the contracts in the second-stage, $n_{2j} = \overrightarrow{v_j} - max\left\{0, \underline{v_j}\right\}$.

Here, we consider the general case where $\underline{v_j} > 0$. Correspondingly, the second-stage optimization problems of high-competency GPs and low-competency GPs are shown as follows

$$\pi_{2j} = \max_{c_{2j}}\{(\overrightarrow{v_j} - \left(p - e_j - c_{2j}\right))pm(1 + c_{2j}) - \frac{1}{2}{c_{2j}}^2\}.$$

As for the variable c_{2j}, the above functions are concave, i.e., $\frac{\partial^2 \pi_{2j}}{\partial {c_{2j}}^2} = -1 + 2mp < 0$. By using the first-order condition, we get

$$\vec{c_{2j}} = \frac{mp(-1+p-\vec{v}_j-e_j)}{-1+2mp}.$$

We now turn to the first period, in which the high-competency GP has to control its competency disclosure level where the low-competency GP would have no incentive to disclose the pseudo-competency information, i.e., $\vec{\pi_l} > \vec{\pi_h^l}$.

If the low-competency GPs choose to diclose his real competency, then the total utility of patients with a valuation $\overrightarrow{v_l}$ in first-period is equal to that of signing the contract in the second-period, that is

$$\vec{v_l} + e_l + c_{1l} - p = \rho(\vec{v_l} + c_{2l} + e_l - p).$$

The corresponding of the valuation $\overrightarrow{v_l}$ can be rewritten as follows

$$\vec{v_l} = \frac{p(1-\rho+m(p(-2+\rho)+\rho))+(-1+2mp)c_{1l}+(-1-mp(-2+\rho)+\rho)e_l}{1+mp(-2+\rho)-\rho}.$$

By substituting the outcome of $\overrightarrow{v_l}$ into the following objective function of the low-competency GPs in the first period

$$\vec{\pi_{1l}} = \max_{c_{1l}}\{(1 - \overrightarrow{v_l})pm(1 + c_{1l}) - \frac{{c_{1l}}^2}{2} + \rho\vec{\pi_{2l}}\},$$

we can prove that the function is concave, i.e., $\frac{\partial^2 \pi_{1l}}{\partial {c_{1l}}^2} < 0$. Then, according to the first-order condition, we can calculate

$$\vec{c_{1l}} = \frac{mp(-m^2p^3(-2+\rho)^2+2(-1+\rho)^2+2mp^2(2+m(4-3\rho)-3\rho+\rho^2)+A_{11})}{(-1+\rho)^2+2m^3p^3(-4+3\rho)+m^2p^2(12-11\rho+\rho^2)-2mp(3-4\rho+\rho^2)},$$

where $A_{11} = -p(-1 + \rho)(-1 + \rho + m(-8 + 3\rho)) + (1 + mp(-2 + \rho) - \rho)^2 e_l$.

By substituting the outcome of $\vec{c_{1l}}, \vec{v_l}$ into the c_{2l}, we can obtain

$$\vec{c_{2l}} = \frac{mp(m^2p^2(8+p(-2+\rho)-6\rho)+(-1+\rho)^2-mp(-1+\rho)(-6+p+\rho)+A_{12})}{(-1+\rho)^2+2m^3p^3(-4+3\rho)+m^2p^2(12-11\rho+\rho^2)-2mp(3-4\rho+\rho^2)},$$

where $A_{12} = mp(-1 - mp(-2 + \rho) + \rho)e_l$.

These outcomes generates that

$$\vec{\pi}_{1l} = (1 - \overrightarrow{v_l})pm(1 + \overrightarrow{c_{1l}}) - \frac{\overrightarrow{c_{1l}}^2}{2} + \rho\left(\left(\vec{v}_l - (p - e_l - \overrightarrow{c_{2l}})\right)mp(1 + \overrightarrow{c_{2l}}) - \frac{\overrightarrow{c_{2l}}^2}{2}\right)\}.$$

If the low-competency GP mimics the high-competency GP, sending the pseudo-competency information to the patients, the threshold is determined by

$$\vec{v}_l^h + e_h + c_{1h} - p = \rho(\vec{v}_l^h + c_{2l}^h + e_h - p),$$

noting that $c_{2l}^h = \frac{mp(-1+p-\vec{v}_l^h-e_h)}{-1+2mp}$. Then we get

$$\vec{v}_l^h = \frac{p(1-\rho+m(p(-2+\rho)+\rho))+(-1+2mp)c_{1h}+(-1-mp(-2+\rho)+\rho)e_h}{1+mp(-2+\rho)-\rho}.$$

The corresponding profit when the low-competency GP discloses Pseudo-competency information is

$$\vec{\pi}_l^h = (1 - \vec{v}_l^h)pm(1 + c_{1l}) - \frac{1}{2}{c_{1l}}^2 + \rho((\vec{v}_l^h - (p - e_l - c_{2l}^h))pm(1 + c_{2l}^h) - \frac{1}{2}{c_{2l}^h}^2).$$

The high-competency GP has to strengthen the competency disclosure intensity to prevent the low-competency GP from imitating, conditional on such disclosure strategy being the most profitable for himself. Consequently, we use the quadratic formula to obtain the condition which guarantees the $\vec{\pi}_l \geqslant \vec{\pi}_l^h$, i.e., ${\vec{c}_{1h}}^{\,b} \leqslant \vec{c}_{1h} \leqslant {\vec{c}_{1h}}^{\,a}$, where

$${\vec{c}_{1h}}^{\,b} = \frac{-b_1+\sqrt{c_1}}{1+\frac{2mp(-1+2mp)}{1+mp(-2+\rho)-\rho}+\frac{m^2p^2(-1+2mp)\rho}{(1+mp(-2+\rho)-\rho)^2}}, \quad {\vec{c}_{1h}}^{\,a} = \frac{-b_1-\sqrt{c_1}}{1+\frac{2mp(-1+2mp)}{1+mp(-2+\rho)-\rho}+\frac{m^2p^2(-1+2mp)\rho}{(1+mp(-2+\rho)-\rho)^2}},$$

and $c_1 = \frac{mp(-1+\rho)(e_l-e_h)(2(m^2p^2(4+p(4-3\rho)-2\rho)+2m^3p^4(-2+\rho)+mp(4+p-\rho)(-1+\rho)+(-1+\rho)^2))}{(1+mp(-2+\rho)-\rho)^2} + \frac{mp(-1+\rho)(e_l-e_h)(mp(2mp-1)(-1+2mp+\rho)e_l-mp(1-2mp)^2(-1+\rho)e_h)}{(1+mp(-2+\rho)-\rho)^2}$, $b_1 = mp(\frac{2mp-1}{1+mp(-2+\rho)-\rho} - 1 + \frac{p(1-\rho+m(p(-2+\rho)+\rho))}{1+mp(-2+\rho)-\rho} - e_r + \frac{\rho((-1+2mp)(-1+mp+\rho)+mp(1+mp(-2+\rho)-\rho)e_g+mp(-1-mp(-2+\rho)+\rho)e_r)}{(1+mp(-2+\rho)-\rho)^2})$. To prevent from being imitated by the low-competency GP easily, the $\vec{c}_{1h}$ should be intensified which satisfy $\vec{c}_{1h} \leqslant {\vec{c}_{1h}}^{\,a}$ (${\vec{c}_{1h}}^{\,b} \leqslant \vec{c}_{1h}$ is excluded). Meanwhile, since the high-competency GP's profit increases with the cmpetency diclosure intensity, they would set $\vec{c}_{1h} = {\vec{c}_{1h}}^{\,a}$ to obtain the maximum profit. Then, for the high-competency GPs, their competency disclosure level in the second stage can be calculated, that is

$$\vec{c}_{2h} = -\frac{mp(-1+\rho+{\vec{c}_{1h}}^{\,a})}{1+mp(\rho-2)-\rho},$$

and the corresponding profit is $\vec{\pi}_h = (1 - \vec{v}_h)pm(1 + {\vec{c}_{1h}}^{\,a}) - \frac{1}{2}{\vec{c}_{1h}}^{\,a2} + \rho\vec{\pi}_{2h}$, which is equivalent to $\vec{\pi}_l$.

Proven.

Proposition 2: In this scenario, the GPs of different type disclose the same level of competency information in the first period, i.e., $\tilde{c}_1 = \tilde{c}_{1h} = \tilde{c}_{1l}$, but the competency disclosure intensity are different in period 2, $\tilde{c}_{2j}, j \in \{h, l\}$. Patients cannot distinguish the GPs' true competency in the first stage, whereas they can accurately tell the GP's type in the second signing stage.

Given the above hypothesis, patients will sign with the specific GP if their valuations are bigger than $\underline{v_j} > p - e_j - c_{2j}$. Then the return function of the low-competency GPs when they do not imitate in the second period becomes

$$\tilde{\pi}_{2l} = \max_{c_{2l}}\{(\tilde{v} - \max\{0, \underline{v_l}\})pm(1 + c_{2l}) - \frac{{c_{2l}}^2}{2}\}.$$

Here, we also pay attention to the general case where $\underline{v_j} > 0$. As for the variable c_{2l}, the above functions are concave (i.e., $\frac{\partial^2\tilde{\pi}_{2l}}{\partial\tilde{c}_{2l}^{\,2}} = -1 + 2mp < 0$), the GP's second-period competency information disclosure intensity is calculated according to the first-order condition, i.e.,

$$\tilde{c}_{2l} = \frac{-mp\tilde{v} - mpe_l - mp + mp^2}{(2mp-1)}.$$

The corresponding profit is

$$\tilde{\pi}_{2l} = (\tilde{v} - (p - e_j - \tilde{c}_{2l}))pm(1 + \tilde{c}_{2l}) - \frac{\tilde{c}_{2l}^{\ 2}}{2}.$$

If the low-competency GPs mimic the high-competency GPs, the lower limit above which patients will sign in the second period becomes $\underline{v_l^h} = p - e_h - c_{2h}$. The corresponding profit after imitating is

$$\pi_{2l}^h = (\tilde{v} - (p - e_h - c_{2h}))pm(1 + c_{2h}) - \frac{1}{2}c_{2h}^{\ 2}.$$

The high-competency GPs should set a level of competency disclosure c_{2h} to ensure that the low-competency GPs have no incentive to imitate. Thus, c_{2h} should satisfy $\vec{\pi}_{2l} > \vec{\pi}_{2l}^h$ and conditional on this, the high-competency GPs extract the maximum profit. Therefore, c_{2h} should satisfy $c_{2h} \geqslant \tilde{c}_{2h}^a$, $c_{2h} \leqslant \tilde{c}_{2h}^b$, where

$$\tilde{c}_{2h}^a = \frac{-mp(mp - \tilde{v} - e_h - 1) + \sqrt{mp(e_h - e_l)(2 - 2mp - 2mp^2 + 2mp\tilde{v} + mpe_l + mpe_h)}}{(1-2mp)},$$

$$\tilde{c}_{2h}^b = \frac{-mp\left(mp - \tilde{v} - e_h - 1\right) - \sqrt{mp(e_h - e_l)(2 - 2mp - 2mp^2 + 2mp\tilde{v} + mpe_l + mpe_h)}}{(1-2mp)}.$$

Similarly, the former situation is excluded, for the value of which is significantly greater than 1, and the competency information disclosure level in period 2, i.e., $c_{2h} = \tilde{c}_{2h}^b$, is set by the high-competency GPs to prevent the low-competency GPs disclosing pseudo-competency information easily.

By substituting $\tilde{c}_{2h}^b$ into the following objective function, the high-competency GPs' profit in the second-stage is obtained:

$$\tilde{\pi}_{2h} = (\tilde{v} - (p - e_r - \tilde{c}_{2h}^b))pm(1 + \tilde{c}_{2h}^b) - \frac{1}{2}\tilde{c}_{2h}^{b\ 2}.$$

Next, we solve the first-stage problem, in which two types of GPs disclose the same competency information. Firstly, we need to compute the following equation to get the threshold $\tilde{v}$:

$$\tilde{v} + \tilde{c}_1 + E[e] - p = \rho(\tilde{v} + E[c_2] + E[e] - p),$$

where $E[c_2] = \gamma\tilde{c}_{2l} + (1-\gamma)\tilde{c}_{2h}^b$ and $E[e] = \gamma e_h + (1-\gamma)e_l$, Hence, the value of $\tilde{v}$ can be calculated as follows

$$\tilde{v} = \frac{(\Psi_1 + \Psi_3\tilde{c}_1 + (\rho-1)^2(p - \gamma e_h) + \sqrt{mp\gamma^2\rho^2(e_l - e_h)(-2\Psi_3\tilde{c}_1 + \Psi_2)})}{(1 + mp(\rho-2) - \rho)^2},$$

where $\Psi_1 = mp(mp^2(\rho-2)^2 + \rho - \rho^2 + p((6 - 2m + \gamma)\rho + (m-2)\rho^2 - 4)) - m^3p^3\gamma(\rho-2)\rho - mp^2\gamma\rho(\rho - m(p(\rho-2) + \rho - 1)) + mp\gamma(2\Psi_4 + mp(4\rho + (\gamma-1)\rho^2 - 4))e_h - 2mp\Psi_4(\gamma-1)e_l + ((\gamma-1)(\rho-1)^2 - m^2p^2((1-\gamma)(\rho-2)^2 + \gamma^2\rho^2))e_l$, $\Psi_2 = 2m^2p^3\gamma\rho(\rho - 1 - mp(\rho-2)) + mp((1-2\gamma)e_l((\rho-1)^2 - 2mp\Psi_4) + e_l(2\gamma-1)(\rho-1)^2e_h + m^2p^2((\rho-2)^2(1-2\gamma) + \gamma^2\rho^2)) - m^3p^3(2\gamma\rho^2 - 8 - 2(\gamma-2)\rho + ((\rho-2)^2(1-2\gamma) + \gamma^2\rho^2)e_h) + mp(10 - 16\rho + 6\rho^2) - 2(\rho-1)^2 + 2m^4p^4\gamma(\rho-2)\rho - 2m^2p^2(8 - 9\rho + 2\rho^2 + (2\gamma-1)\Psi_4e_h)$, $\Psi_3 = (2mp-1)(1 + mp(\rho-2) - \rho)$ and $\Psi_4 = 2 - 3\rho + \rho^2$.

Then, based on the following profit maximization, the GPs determines their first-period disclosure intensity:

$$\tilde{\pi} = \max_{\tilde{c}_1}\{(1-\tilde{v})pm(1 + \tilde{c}_1) - \frac{1}{2}\tilde{c}_1^{\ 2} + \rho(\gamma\tilde{\pi}_{2h} + (1-\gamma)\tilde{\pi}_{2l})\}.$$

As easily can be seen, even with this simplification, the value of $\tilde{c}_1$ is still difficult to be analytically calculated, hence, numerical simulation methods are used to verify and solve it..

Finally, the total profits of each GP are solved by substituting $\tilde{c}_1$ into these objective functions:

$$\tilde{\pi}_h = (1-\tilde{v})pm(1+\tilde{c}_1) - \frac{1}{2}\tilde{c}_1^{\,2} + \rho\tilde{\pi}_{2h}, \tilde{\pi}_l = (1-\tilde{v})pm(1+\tilde{c}_1) - \frac{1}{2}\tilde{c}_1^{\,2} + \rho\tilde{\pi}_{2l}.$$

Proven.

Proposition 3: In this scenario, the GPs of different types disclose the same level of competency information in each period, i.e., $\hat{c}_1 = c_{1j}$, $\hat{c}_2 = c_{2j}$, $j \in \{h, l\}$. Patients cannot distinguish the GP's true type in the first stage by analyzing the information they receive, but in the second period, prospective patients can infer the GP's type by assessing the signal about the GP from early sufferers. Hence, these patients will update their expectations about the GPs' competency when they receive this signal.

The problem consists of two periods, we solve the problem backward. Suppose the threshold is $\hat{v}$, below which sufferers would delay signing in the stage 2. Patients might receive two kinds of signals, namely, s_h and s_l. Given that the lowest valuation above which patients will sign is

$$\underline{v}(s_h) > p - E[e|s_h] - \hat{c}_2,$$

the value of which is presumed to be nonnegative.

The optimization problem of the GPs in this scenario reads as:

$$\pi_{2j}(s_h) = \max_{\hat{c}_2}\{(\hat{v} - (p - E[e|s_h] - \hat{c}_2))pm(1+\hat{c}_2) - \frac{1}{2}\hat{c}_2^{\,2}\},$$

which is concave with respect to $\hat{c}_2$. Thus, according to the first-order condition, we can calculate that

$$\hat{c}_2(s_h) = \frac{mp(-1+a+p-ap+\gamma-2a\gamma-p\gamma+2ap\gamma+(-1+a+\gamma-2a\gamma)\hat{v}+(-1+a+\gamma-a\gamma)e_l-a\gamma e_h)}{(-1+2mp)(1-\gamma+a(-1+2\gamma))}.$$

The corresponding profit $\pi_{2h}(s_h) = \pi_{2l}(s_h) = (\hat{v} - (p - E[e|s_h] - \hat{c}_2))pm(1+\hat{c}_2) - \frac{1}{2}\hat{c}_2^{\,2}$ is obtained by substituting $\hat{c}_2(s_h)$ into the profit function.

Similarly, when the patients get the signal s_l, the corresponding threshold above which patients sign the contract with the GP is

$$\underline{v}(s_l) > p - E[e|s_l] - \hat{c}_2,$$

which is also presumed to be nonnegative.

The GP decide the disclosure intensity $\hat{c}_2$ by computing the problem

$$\pi_{2j}(s_l) = \max_{\hat{c}_2}\{(\hat{v} - (p - E[e|s_l] - \hat{c}_2))pm(1+\hat{c}_2) - \frac{1}{2}\hat{c}_2^{\,2}\},$$

which yields that

$$\hat{c}_2(s_l) = \frac{mp(a-ap+\gamma-2a\gamma-p\gamma+2ap\gamma+(a+\gamma-2a\gamma)\hat{v}+(a-a\gamma)e_l+\gamma e_h-a\gamma e_h)}{(-1+2mp)(-\gamma+a(-1+2\gamma))}.$$

Substituting for $\hat{c}_2(s_l)$ in the profit function, the corresponding profit $\pi_{2h}(s_l) = \pi_{2l}(s_l)$ can be obtained.

Focusing on the first period, the threshold $\hat{v}$ is determined by

$$\hat{v} + \hat{c}_1 + E[e] - p = \rho(\hat{v} + E[c_2] + E[e] - p),$$

where $E[c_2] = \Pr(s_h)\hat{c}_2(s_h) + \Pr(s_l)\hat{c}_2(s_l)$. Thus, we can calculate

$$\hat{v} = \frac{p-2mp^2-\mu+2mp\mu-p\rho+mp\rho+mp^2\rho+\mu\rho-2mp\mu\rho+(-1+2mp)\hat{c}_1-mp(\gamma-1)\rho e_l+mp\gamma\rho e_h}{1+mp(-2+\rho)-\rho},$$

The GP determines the first-period disclosure intensity to maximize the following total discounted revenue function:

$$\hat{\pi} = \max_{\hat{c}_1}\{(1-\hat{v})pm(1+\hat{c}_1) - \frac{1}{2}\hat{c}_1^{\,2} + \rho(\Pr(s_h)\pi_{2j}(s_h) + \Pr(s_l)\pi_{2j}(s_l))\}.$$

Because we can not compute the explicit solution, numerical analysis method is adopted to verify the equilibrium disclosure effort accordingly.

Proven.
